# Regulation of Ras Exchange Factors and Cellular Localization of Ras Activation by Lipid Messengers in T Cells

**DOI:** 10.3389/fimmu.2013.00239

**Published:** 2013-09-04

**Authors:** Jesse E. Jun, Ignacio Rubio, Jeroen P. Roose

**Affiliations:** ^1^Department of Anatomy, University of California San Francisco, San Francisco, CA, USA; ^2^Institute for Molecular Cell Biology, Center for Sepsis Control and Care (CSCC), University Hospital, Friedrich-Schiller-University, Jena, Germany

**Keywords:** T cell, signaling, lipids, Ras, SOS, RasGRP, LAT, P38

## Abstract

The Ras-MAPK signaling pathway is highly conserved throughout evolution and is activated downstream of a wide range of receptor stimuli. Ras guanine nucleotide exchange factors (RasGEFs) catalyze GTP loading of Ras and play a pivotal role in regulating receptor-ligand induced Ras activity. In T cells, three families of functionally important RasGEFs are expressed: RasGRF, RasGRP, and Son of Sevenless (SOS)-family GEFs. Early on it was recognized that Ras activation is critical for T cell development and that the RasGEFs play an important role herein. More recent work has revealed that nuances in Ras activation appear to significantly impact T cell development and selection. These nuances include distinct biochemical patterns of analog versus digital Ras activation, differences in cellular localization of Ras activation, and intricate interplays between the RasGEFs during distinct T cell developmental stages as revealed by various new mouse models. In many instances, the exact nature of these nuances in Ras activation or how these may result from fine-tuning of the RasGEFs is not understood. One large group of biomolecules critically involved in the control of RasGEFs functions are lipid second messengers. Multiple, yet distinct lipid products are generated following T cell receptor (TCR) stimulation and bind to different domains in the RasGRP and SOS RasGEFs to facilitate the activation of the membrane-anchored Ras GTPases. In this review we highlight how different lipid-based elements are generated by various enzymes downstream of the TCR and other receptors and how these dynamic and interrelated lipid products may fine-tune Ras activation by RasGEFs in developing T cells.

## Non-Oncogenic Ras Activation First Observed in T Lymphocytes

**Figure 1:**
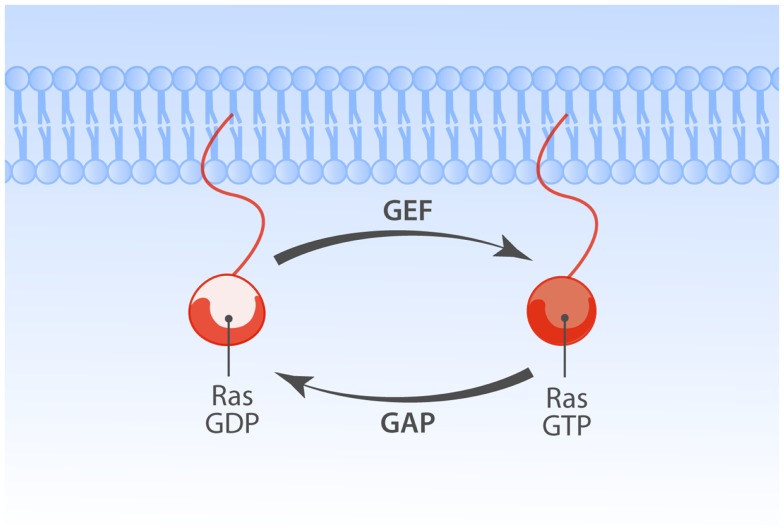
**Regulation of Ras family proteins**. The Ras GTPases cycle between GDP-bound inactive and GTP-bound active forms. Activation of Ras is regulated by the balance of opposing actions of two classes of Ras regulatory enzymes. Guanine nucleotide exchange factors (GEFs) promote GTP-bound Ras state by enhancing exchange of GDP with GTP. GTPase activating proteins (GAPs) enhance slow rate of intrinsic Ras GTPase activity, promoting the inactive GDP-bound state of Ras.

Ras is a membrane-bound small GTPase that plays a pivotal role in transducing responses to diverse extracellular signals that impact various cellular processes, prominently cell proliferation, differentiation, apoptosis (1). Ras cycles between a GTP-associated active state (Ras·GTP) and GDP-bound inactive state (Ras·GDP). In both the Ras·GDP and Ras·GTP states the nucleotide is very tightly bound (2–4) and for Ras activation to occur Ras guanine nucleotide exchange factors (RasGEFs) need to loosen the grip of Ras on the bound nucleotide, stabilizing nucleotide-free Ras that stochastically but preferentially associates with GTP, because GTP is present in the cell in higher concentrations than GDP (5). Reciprocally, GTP hydrolysis is critical for inactivation from Ras·GTP to Ras·GDP and Ras’ modest intrinsic rate of GTP hydrolysis requires the hydrolysis-augmenting action of RasGAPs (Ras GTPase activating proteins) (Figure 1).

The physiological importance of Ras’ GTPase activity was recognized in the late 80s through the detection and biochemical characterization of GTPase impairing Ras mutations commonly found in various human tumor tissues (6). Ras·GTP is a potent signaling hub, connecting to many downstream effector molecules like RAF, PI3K, and RalGDS. The best-characterized signaling cascade is the Ras·GTP-RAF-MEK-ERK pathway (4, 7, 8). In cells without mutations in Ras only a small portion of the total amount of Ras is GTP-loaded following receptor stimuli, which makes detection more challenging. In the early 90s Doreen Cantrell’s group first showed Ras activation (or Ras·GTP loading) in normal T lymphocytes that were stimulated with the interleukin 2 (IL2) cytokine or a phorbol ester, agents that were known to induce lymphocyte proliferation (9, 10). The physiological significance of biochemical signals transduced by an intact Ras-RAF-MEK-ERK pathway in lymphocytes was subsequently shown through transgenic expression of mutant Ras- and MEK-alleles in thymocytes; for example, expression of dominant-negative H-Ras^S17N^ under the control of *lck* promoter or catalytically inactive MEK-1 perturbs positive selection of developing thymocytes (11, 12).

Research over the past two decades has revealed many intricate ways of regulated Ras activation, not only in lymphocytes but also in other cell types. In this review we will discuss the role of lipid messengers in regulating the Son of Sevenless (SOS) and RasGRP RasGEF families. We will focus on recent findings related to lipid-RasGEF regulation, recent insights from novel mouse models, as well as on the ongoing debate of the cellular compartment or location of Ras activation. For additional information on the RasGEF family of exchange factors we refer to previous review articles (8, 13–15).

## The Players; Three Families of Ras Guanine Nucleotide Exchange Factors

The earlier-mentioned dominant-negative Ras approach established a critical role for Ras in lymphocytes. Data from numerous laboratories have meanwhile demonstrated that dominant-negative Ras^S17N^ exerts its blocking action mainly by usurping and blocking RasGEFs [although other features of Ras^S17N^ probably contribute to its inhibitory action (16, 17)]. Thus, the ability of dominant-negative Ras^S17N^ to affect lymphocyte biology not only highlights the importance of Ras but points also to a critical role of GEFs.

**Figure 2:**
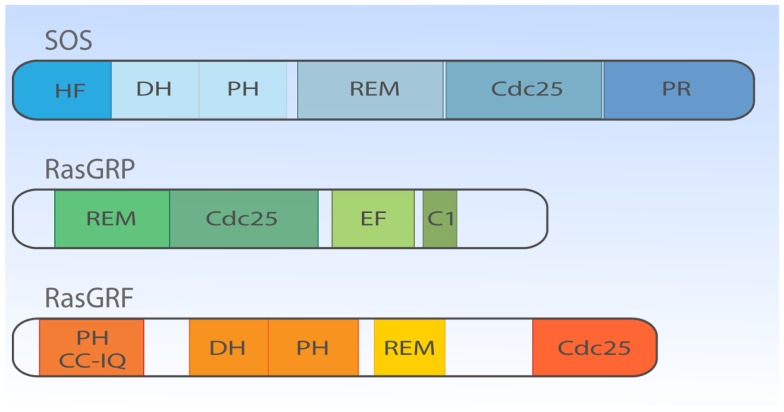
**Structural domain organization of three families of RasGEFs expressed in T cells**. Cartoon highlighting the general protein domains in the three families of RasGEFs: SOS, RasGRP, and RasGRF. Cdc25, Cdc25 homology domain; DH, Dbl homology domain; HF, N-terminal histone-like fold; PH, Pleckstrin homology domain; PR, C-terminal PR domain; REM, Ras exchange motif; EF, Ca^2+^-binding EF hand; C1, DAG-binding C1 domain; CC-IQ, coiled coil – ilimaquinone domain. Protein size is drawn to approximate scale based on SOS1, RasGRP1, and RasGRF1 (53).

If we fast-forward roughly two decades, we now know that lymphocytes can simultaneously express three types of RasGEF proteins (Figure 2). The overlapping expression profiles create the impression of seemingly redundant and unnecessary complex mechanisms to couple antigen receptor stimulation to Ras activation. However, distinct lymphocyte developmental defects in mice deficient for unique RasGEFs argue for specialized functions for each RasGEF (18–20). We will cover the mouse phenotypes in more detail in subsequent paragraphs and will first focus on the different protein domains in the three RasGEF families [also reviewed in Ref. (5, 8)].

### Son of sevenless

**Figure 3:**
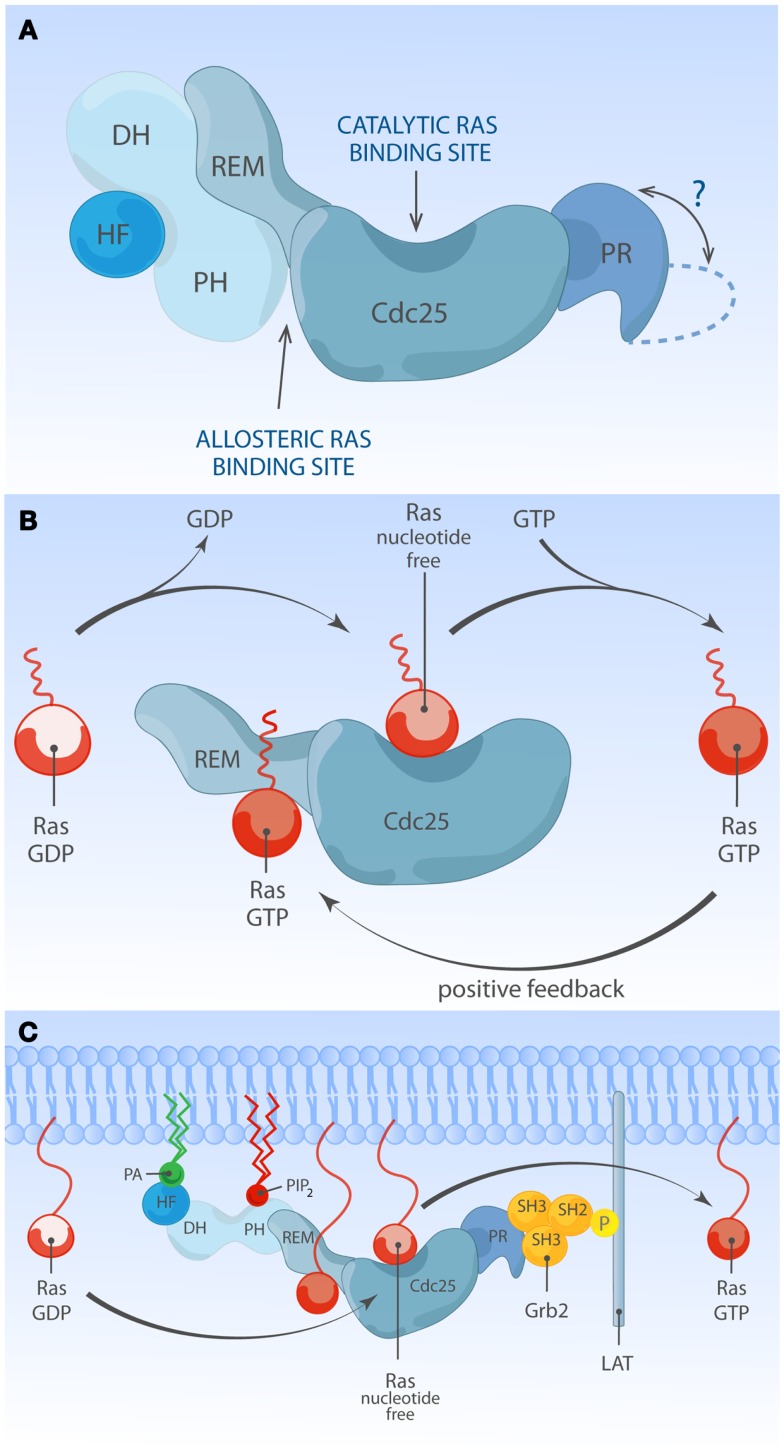
**Multiple membrane-derived signals determine the RasGEF activity of SOS**. **(A)** Model of inactive SOS. In the inactive state, SOS’s DH-PH domains obscure the allosteric Ras-binding pocket. Without engagement of the allosteric pocket by Ras·GTP, SOS only shows low reactivity for Ras·GDP at the catalytic binding site. The HF docks itself to a helical linker region (not depicted) between PH and REM domains, further stabilizing auto-inhibited state of SOS. The protein structure of the C-terminal proline-rich domain has not been determined to date. **(B)** Model of allosteric activation of SOS. Ras·GTP binding to the allosteric site enhances SOS exchange activity by increasing Ras-binding affinity for the catalytic pocket, establishing a positive feedback mechanism. Other SOS domains are omitted for simplicity. **(C)** Model of full activation of SOS. Full activation of SOS requires the integration of multiple membrane-derived signals. Grb2-mediated membrane recruitment of SOS to phosphorylated LAT is thought to be one of the initial membrane recruitment mechanisms. Membrane phospholipids such as PIP_2_ and PA interact with HF and PH domains, and these interactions further relieve auto-inhibition state of SOS, allowing efficient access of Ras to both the allosteric and catalytic sites.

There are two members in SOS-family RasGEFs, SOS1 and SOS2. Structurally, the SOS protein is composed of six domains that have distinct functional importance: starting from the N-terminus, the histone-like fold (HF), the Dbl homology domain (DH), the Pleckstrin homology (PH) domain, the Ras exchange motif (REM), the Cdc25 homology domain, and the proline-rich (PR) domain (Figures 2 and 3). The naming of HF comes from structural resemblance to histone 2 dimer H2a-H2b, and HF mediates lipid interaction with phosphatidylinositol 4,5-bis phosphate [PI(4,5)P_2_, hereafter PIP_2_] or phosphatidic acid (PA) (21). The DH domain is a functional domain commonly found in Rho family GEFs, suggesting SOS may also have Rho-specific GEF function in addition to the more established RasGEF activity (22, 23). PH domains are lipid/protein-interacting domains (24). The PH domain of SOS has an auto-inhibitory function, that is regulated by interaction with membrane lipids such as PIP_2_ or PA (25–29). REM-Cdc25 domains make up the RasGEF catalytic core of SOS and all other RasGEFs. Unique to SOS, its catalytic core contains two distinct Ras-binding sites: one for GDP/GTP exchange and the other for allosteric regulation of SOS by Ras (30, 31). The C-terminal PR domain is the only segment of SOS that remains to be structured for analysis. Functionally, the PR domain contains multiple PR motifs that can bind SH3 domain-containing proteins such as the SH2-SH3-SH2 adapter Grb2 (32, 33), the p85 subunit of PI3kinase (34), PLCγ1 (35–38), and Avi1/E3b1 (39). In addition, the PR domain contains multiple documented phosphorylation sites of ERK and probably other kinases (40–44), spiked in between the PR stretches that are, at least in part, postulated to play a role in feedback control of SOS activity.

### Ras guanine nucleotide releasing proteins

**Figure 4:**
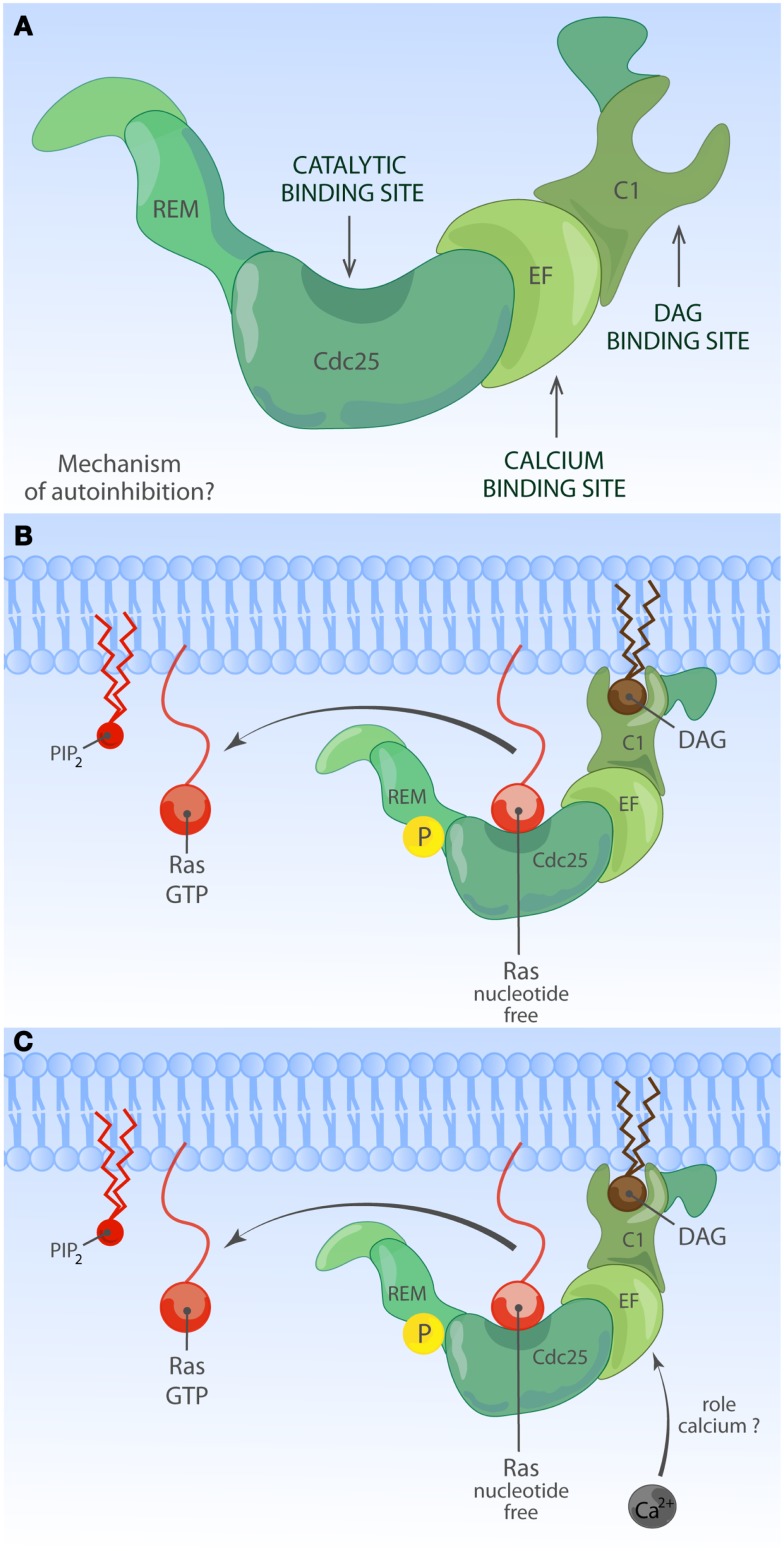
**Activation of RasGRP**. **(A)** Depiction of RasGRP with its protein domains. RasGRPs must be controlled to prevent spurious Ras activation but the exact mechanism of auto-inhibition is unknown. Roles for various domains C-terminal of the Cdc25 domain to limit membrane recruitment of RasGRP have been proposed. **(B)** DAG-regulated membrane recruitment of RasGRP. Receptor-induced generation of diacylglycerol (DAG) results in efficient membrane recruitment on RasGRP1, RasGRP3, and RasGRP4 where these RasGEFs can encounter Ras·GDP to activate it to Ras·GTP. RasGRP1 and RasGRP3 are known to be phosphorylated on a conserved threonine residue at the very start of the Cdc25 domain, which enhances their catalytic activity through an unknown mechanism. RasGRP2 does not efficiently bind DAG and must have a different membrane-recruiting mechanism. **(C)** Other regulatory mechanisms for RasGRP. Amino acid sequence homologies predict that RasGRPs lack and allosteric Ras-binding pocket as the one observed for SOS. RasGRP proteins contain EF hands, structure that can often bind calcium. Calcium has been implicated in the recruitment of RasGRP1 to the membrane but nuances appear to exist in different cell types. It is not known if other lipid moieties such as PIP_2_ can regulate the activity or residence time of RasGRP1 at the membrane.

Much less is known about the function of the domains or even the identity of domains in the RasGRP RasGEFs. To date, there is no RasGRP structure and we are therefore limited to make predictions based on amino acid sequence. There are four RasGRP proteins, RasGRP-1 through RasGRP-4, with specific expression profiles and nuances in biochemical function. All RasGRP’s contain a central catalytic core consisting of the catalytic REM-Cdc25 cassette. Sequence divergency between the RasGRP and SOS REM-Cdc25 cores predicts that RasGRPs are not regulated through an allosteric activation mechanism. Although RasGRP2 contains the REM-Cdc25 core and early studies indicated RasGEF activity (45), it is generally accepted that RasGRP2 functions as a GEF for the small GTPase Rap (46). Analogously, all four proteins are predicted to have a C1 domain positioned C-terminal of the catalytic core, but again, RasGRP2 appears to be most divergent in that its C1 domain does not bind diacylglycerol (DAG) (47) and RasGRP2 protein does not translocate to the membrane when cells are stimulated with DAG analogs (48). A third shared domain in all RasGRP proteins is the pair of EF hands that occupies an interesting position in the protein, sandwiched between the catalytic core and the C1 domain (Figures 2 and 4). EF hands typically come in pairs with each hand binding one calcium ion (49, 50). However, not all EF hands bind calcium. For instance, RasGRP1 with two predicted EF hands based on the amino acid sequence can only bind one calcium ion with one EF hand, not with both (51). Close examination of the sequence similarities and divergence in the EF hand domains of all RasGRP proteins (not shown) tells us that there are likely going to be substantial differences in the ways that the different RasGRP’s are regulated by calcium. Thus, the four RasGRP proteins demonstrate specific biochemical regulatory mechanisms and activities that have likely evolved over time to establish their individual exchange functions in the specific cell types where they are expressed. In this review we will not cover the differences between the RasGRPs in much more detail, instead we refer you to an excellent review by Stone (15) and one on cancer (52).

### Ras guanine nucleotide releasing factor

More closely related to SOS than RasGRP are RasGRF’s; RasGRF-1 and RasGRF-2 make up this family of proteins with multiple domains [reviewed in Ref. (53)]. Similar to the two other RasGEF family proteins, RasGRF proteins contain a REM-CDC25 catalytic core domain. Uniquely, RasGRFs contain two PH domains; one at the N-terminus (PH1) and the other PH in tandem with the DH domain (PH2), similar to the configuration of the DH-PH domain of SOS-family proteins (Figure 2). PH1 cooperates to promote stimulation-dependent membrane localization of RasGRF in fibroblasts, probably through interaction with membrane lipid (53–55). The coiled-coil (CC) domain is known to mediate protein oligomerization (56), whereas the ilimaquinone (IQ) domain mediates calmodulin binding (57). In cooperation with the PH1 domain, CC and IQ domains notably mediate the interaction with a MAPK p38 scaffold protein IB2/JIP2 in COS7 cells (58), which is interesting because the DH-PH domain of RasGRF has GEF activity toward Rac (59, 60) indicating that RasGRF may efficiently link Rac to the p38 pathway through the IB2/JIP2 scaffold protein (58).

### Expression patterns of the eight RasGEF genes

The RasGRP and RasGRF families of exchange factors have tissue-specific expression patterns whereas SOS proteins are ubiquitously expressed (15). For instance, RasGRP1 is expressed in dynamic patterns in developing T cells (20, 61), in the brain (46), and in primary keratinocytes (62). RasGRF1 and RasGRF2 are predominantly expressed in the central nervous system (63). In addition, RasGRF2, but not RasGRF1, is expressed in T cells (64). Analyses of *rasgrf2*-deficient mice revealed that this RasGEF play a critical role in the activation of NFAT target genes in T cells (64). However, T cell development is normal in *Rasgrf2^−/−^* mice, and Rasgrf2 appears to have only limited activity toward Ras-ERK in T cells (64). We will therefore limit ourselves to the regulation of SOS and RasGRP here. Significantly, these two distinct types of RasGEFs cooperate to establish robust yet controlled activation of Ras and Ras’ RAF-MEK-ERK effector pathway (65, 66). In response to T cell receptor (TCR) stimulation, both RasGRP1 and SOS are recruited to the membrane where they encounter membrane-anchored Ras and both convert Ras·GDP to Ras·GTP. Why is it then that knockout mouse models for SOS1 and RasGRP1 show different impairments in terms of thymocyte selection and T cell development (20, 61, 67)?

### Auto-inhibition of SOS RasGEFs

Ample structural and cellular studies indicate that catalytic activity of SOS1 is self-limited by an intramolecular auto-inhibitory mechanism which involves multiple internal protein domains. Auto-inhibition can be relieved by membrane signals from proteins and lipid species. The physiological relevance of auto-inhibition of SOS1 is highlighted by a clinical condition called Noonan syndrome (NS). NS is a relatively common autosomal developmental abnormality and RASopathy, a disease that is caused by germ-line mutations in molecules leading to modestly increased Ras signaling (68, 69). NS is genetically heterogeneous: the majority of mutations are associated with PTPN11, K-Ras, N-Ras, SOS1, B-Raf, Raf-1, SHOC1, and CBL (69). Among eight NS-associated genes, missense mutations in SOS1 are identified in about 10% of NS cases (69–73). Most NS-associated SOS1 mutations are predicted to relieve auto-inhibiting structural constraints within SOS1, allowing increased signal output through the Ras pathway. Indeed, several NS-associated *SOS1* mutant alleles (R552G, E108K, W729L, and E846K) have been experimentally characterized *in vivo*, showing increased Ras·GTP accumulation and ERK activation at basal state or upon stimulation (70, 71, 74, 75). These findings visibly illustrate that normal SOS1 function is tightly regulated and highlight the clinical relevance of such regulation (Figure 3A). These observed defects in fine-tuning of Ras activity control in NS cells are also likely to impact on the patient’s immune biology, because patients with gain-of-function mutations in Ras proteins are at a higher risk of developing autoimmune disorders (76–79). In the following few sections, we will review the literature on normal SOS regulatory mechanisms and how membrane-based signals from proteins and phospholipids influence the activation status of SOS.

## Membrane Recruitment of SOS by Grb2: Initial Step in SOS Activation

T cell receptor stimulation leads to rapid activation of Src family kinases and the Syk family kinase ZAP70. ZAP70 phosphorylates the adapter LAT, a key scaffold to which various downstream signal transducers are assembled, including molecules that are coupled to Ras-MAPK pathway activation (80). Prior to cell stimulation, most SOS is found in the cytoplasmic compartment, constitutively bound to the SH3-SH2-SH3 domain-containing adapter Grb2. Upon stimulation, SOS rapidly localizes to the plasma membrane (PM) (32, 33, 81, 82). SOS1 membrane targeting is an essential event for SOS-Ras activation and is mediated by binding of the SH2 domain of Grb2 (with SOS1) to phosphorylated tyrosine residues of LAT (82). A truncated SOS1 variant incapable of Grb2 binding is still functional as a RasGEF but can activate Ras only if targeted elsewise to the membrane, indicating that membrane recruitment is an essential step in ligand-dependent activation of SOS (83). Unlike Ras, lipid modification of SOS was never been reported. Therefore, Grb2-mediated membrane anchorage has been viewed as the key regulatory mechanism of SOS GEF signal output.

However, the traditional view that Grb2 association is dominant or even essential for SOS1 membrane targeting has also been challenged. Expression of C-terminally truncated SOS1 incapable of Grb2 binding has been documented to have comparable or even better Ras-ERK signal responses compared to full-length SOS1 (84–86). Similarly, SOS^ΔC^, a C-terminally truncated SOS mutant lacking residues 1050–1333 becomes recruited to the membrane in response to serum stimulation, indicating that Grb2 is not the only mechanism for ligand-dependent SOS1 membrane targeting (29). These studies may collectively imply that Grb2 is a redundant mechanism for stimulation-dependent SOS membrane localization and subsequent SOS activation. However, little attention is given to the physiological relevance of the protein levels of the C-terminal truncated SOS1 variant examined in these studies and time kinetics of Ras-ERK response. It is very plausible that Grb2 is important and a major membrane anchorage mechanism when physiological levels of SOS1 are available to the activated ligand. Supporting this notion, structural studies and recent mouse embryonic stem cell (mESC) study demonstrate that, besides Grb2-mediated membrane recruitment, the SOS1 activity is determined by summation of weak to moderate membrane protein and lipid interactions mediated by multiple protein domains of SOS1 (87).

## Allosteric Activation of SOS; a Positive Feedback Loop

The SOS1-mediated nucleotide exchange rate on Ras is 500-fold higher when Ras is membrane-bound compared to when Ras activation is measured in solution (88), supporting a view that ligand-dependent membrane recruitment of SOS1 not only exists to promote the chance of substrate encounter but is also instrumental to enhance SOS1 enzymatic activity. One hint for the existence and identity of additional membrane signals regulating SOS1 came from structural studies by the Bar-Sagi and Kuriyan groups. Unexpectedly SOS1 was found to be associated with two discrete Ras molecules, forming a 2:1 ternary complex between two Ras molecules and one SOS1 molecule. One Ras molecule serves as a substrate and is bound at its catalytic pocket within the Cdc25 domain, while the second non-substrate Ras occupies the allosteric site in the REM domain (31). Occupation of the allosteric site by Ras·GTP results in conformational change stabilizing SOS1 catalytic pocket and stimulates *in vitro* nucleotide exchange activity by ∼75-fold (89, 90). In support of this notion, a SOS1 mutant unable to bind to Ras at allosteric site (W729E) shows reduced affinity for Ras at the catalytic site and has low *in vitro* activity (89). The allosteric Ras-binding pocket shows 10-fold higher affinity for GTP-loaded Ras than Ras·GDP. This preferential affinity for Ras·GTP endows SOS1 to sense the activation status of Ras at the membrane and establishes a positive feedback regulation (Figure 3B) (31, 91). Ectopic expression studies provided *in vivo* evidence of allosteric regulation of SOS1 in COS-1 cells (89, 91) or Jurkat cells (65, 66). Recently, allosteric mutant-SOS1 reconstitution into SOS-deficient mESC (87) and DT40 B cells (92) provided more definitive proof of allosteric SOS1 activation regulating the output through the Ras-ERK pathway. In addition to enhancing catalytic activity of SOS, allosteric Ras·GTP binding could potentially affect SOS residence time at the PM by providing an additional membrane anchor for SOS1 other than Grb2 binding.

## Regulation of SOS by Membrane Lipids

Current evidence argues that allosteric Ras binding to SOS1 is such a pivotal step that SOS stays inactive unless Ras·GTP is bound at the allosteric site (93). Then, how has SOS1 evolved to limit spontaneous signaling yet allow for controlled allosteric activation near the membrane interface? In this regard, N-terminal SOS domains play a critical role in regulating SOS1 activation in the context of membrane proximity by sensing membrane lipids.

One membrane lipid sensing N-terminal regulatory unit is the tandem DH and PH domain. *In vitro* and *in vivo* studies identified DH-PH domain being important for membrane-proximal SOS regulation (29, 84, 87, 93). DH domain is commonly found with GTP exchange factors (discussed later). In SOS, the DH domain serves as a gatekeeper preventing promiscuous access to the allosteric Ras-binding pocket. In its auto-inhibited state, SOS1 DH domain blocks the allosteric pocket from Ras binding, which has a critical impact on SOS1’s catalytic pocket. Without allosteric activation the catalytic pocket is not fully receptive to accommodate Ras·GDP and the helical hairpin of SOS1 is not in the correct orientation to dislodge GDP from Ras (89, 93). PH domain is generally known for protein or lipid interactions (55). The PH domain of SOS1 was shown to have affinity for PIP_2_ (25–28) or PA (29). The auto-inhibiting DH domain can be released by electrostatic interaction of membrane PIP_2_ or PA with positively charged residues within the PH domain (29, 93). Therefore, lipid-DH-PH interactions facilitate re-orientation of SOS1 at the membrane interface, allowing allosteric Ras binding (Figure 3C). In support, addition of cell-permeable PA to COS-1 cells is sufficient to induce GTP loading of Ras, and charge-inversion mutations of H475E and R479E in SOS1 abolish PA interaction and PA-induced Ras·GTP loading response (29). Similarly, two different basic residues (K456 and R459) within the PH domain interact with PIP_2_ (93). The biological significance of PIP_2_-PH domain interaction during mESC differentiation was elegantly demonstrated in a recent report from Tony Pawson’s group (87).

Located upstream of DH-PH domains, the HF is an evolutionarily conserved segment (residue 1–191) resembling dimerized histone (21). Based on structural studies, this HF docks itself into the helical linker region of SOS1, located between DH-PH domains and catalytic segment (REM-Cdc25), ensuring SOS auto-inhibition by blocking allosteric activation and by stabilizing a closed conformation of SOS (88, 94). HF interacts with membrane lipids such as PA and PIP_2_, and HF-lipid interaction reverses auto-inhibitory docking, allowing allosteric and catalytic Ras binding at distal and proximal Ras-binding sites (75, 88). Electrostatic charge distribution at the phospholipid-interacting interface of HF appears to be finely tuned by charge neutralization, e.g., the negatively charged residue E108 is surrounded by patches of basic residues (75). Disturbing charge balance by offsetting positive charges leads to reduced Ras-ERK activation in COS-1 and mouse ES cells (75, 87). Additionally, a negative charge neutralization mutation (E108K) is found to be associated with a hyperactive SOS1 allele of human NS (71, 75).

There are some inconsistencies in the lipid species recognized by SOS1’s N-terminal regulatory domains (29, 75, 88, 93). This discrepancy might arise from the variability in the presence of regulatory domains or post-translational modifications of the SOS1 proteins investigated. Perhaps more significant, membrane lipids are also dynamically regulated during cell activation processes (reviewed in Krishna and Zhong (95) in this Research Topic and by Sauer and Cooke (96)). Perhaps, the reported discrepancies regarding the role of lipid species may reflect heterogeneous lipid patterns in distinct cellular backgrounds and the involvement of different lipids at different stage of SOS1 activation.

Taken together, studies *in vitro* and *in vivo* support the view that N-terminal HF and DH-PH domains serve as membrane lipid sensing regulatory segments. On one hand, lipid mediated regulation of SOS1 leads to juxtaposition of SOS1 to substrate/effector. On the other hand, the regulatory domains also contribute to prevent spontaneous activation of SOS1. In this regard, it is worth noting that the second class of human NS-associated SOS1 mutations target N-terminal regulatory domains and often implicate enhanced membrane recruitment of the mutant SOS protein (73).

## RasGRP Auto-Inhibition?

RasGRP proteins have been studied most extensively in T- and B-lymphocytes. In these lymphocytes, RasGRP1 and RasGRP3 activate Ras in a manner that is non-redundant with SOS (18, 65, 97–101). More recently, RasGRP proteins, particularly RasGRP1, have also been associated with human diseases such as autoimmune disease and cancer.

Single nucleotide variants near *RASGRP1* are associated with susceptibility to autoimmune (Type 1) diabetes and to thyroid autoantibodies in Graves disease (102, 103). At this point it is not known what effect these variants in non-coding regions of the *RasGRP1* gene have, but possible mechanisms include altered expression or RasGRP1. *RASGRP1* splice variants have been documented for patients with systemic lupus erythematosus (SLE) (104). Several of these RasGRP1 mRNA splice variants are predicted to miss portions of RasGRP1’s EF hands, which may have an important regulatory role (see below). In addition, it also appeared that many splice variants resulted in lower proteins expression levels of RasGRP1 (104).

RasGRP4 was originally isolated as a Ras activator in acute myeloid leukemia (AML) (105). RasGRP3 plays a role in human melanoma (106) and in prostate cancer (107) that are distinct from those of SOS. When overexpressed from transgenes, RasGRP1 promotes the development of squamous cell carcinoma and melanoma in mouse models in conjunction with skin wounding or carcinogen painting of the skin (108–110). Transgenic over-expression of RasGRP*1* in developing T lymphocytes causes thymic lymphomas in mice (111) and several unbiased mouse model screens for leukemia genes have identified the RasGRP*1* locus as a hot-spot for leukemia virus integrations driving blood cancer (112–114). The molecular basis of these viral integrations is that these cause leukemia through the dysregulated expression of the target gene, typically through overexpression. Significantly, Oki and colleagues as well as our own group have recently shown that elevated RasGRP1 expression also occurs in T cell leukemia patients (115, 116). For more detailed reading on RasGRP1’s role in cancer we refer you to a different review (52). Needless to say these studies collectively indicate that RasGRP1 requires tight regulation. Regulation occurs most definitely at the level of RasGRP1 expression since dysregulated expression of a wild-type RasGRP1 form results in leukemia (116). Extrapolating from our knowledge of SOS1, we propose that RasGRP1 also possesses an auto-inhibited state (Figure 4A) to prevent spurious activation and to balance the activating mechanisms of molecules like DAG, which we will discuss next.

## Diacylglycerol as a RasGRP1 Activator

Phorbol esters such as PMA (a synthetic DAG analog produced out of the plant-derived compound phorbol) had long been known as potent stimulators of Ras activation, but it was not until 1998 when Stone and colleagues cloned the *RasGRP1* gene, that the biochemical connection between DAG and Ras activation was established (51).

In T lymphocytes that receive a TCR stimulus, PLCγ1 is recruited to the membrane and activated so that it cleaves PIP_2_ into inositol-3-phosphate (IP_3_) and DAG. IP3 couples to the calcium pathway (117) and we will come back to this in a moment. The increase of DAG levels in the membrane results in recruitment of RasGRP1 through its C1 domain to the membrane where is can activate Ras (Figure 4B) (19, 51). There is a second, indirect route from DAG to RasGRP1 and RasGRP3, which involves PKC-mediated phosphorylation of these two RasGEFs. RasGRP1 is phosphorylated on threonine 184 (T184) in TCR-stimulated T cells whereas RasGRP3 is phosphorylated on the analogous site, T133, in BCR-stimulated B cells (97, 100, 118). Mutations of T184 or T133 into alanine residues results in impaired, but not absent, stimulus-dependent Ras activation (97, 118) and incubation of cells with PKC inhibitors blocks the phosphorylation of RasGRP1 on T184 (65, 97, 100), providing a rationale for the long established observation that PKC inhibition inhibits the output through the Ras-ERK pathway in lymphocytes. How the phosphorylation of RasGRP1 and RasGRP3 enhances their RasGEF activity is not known.

**Figure 5:**
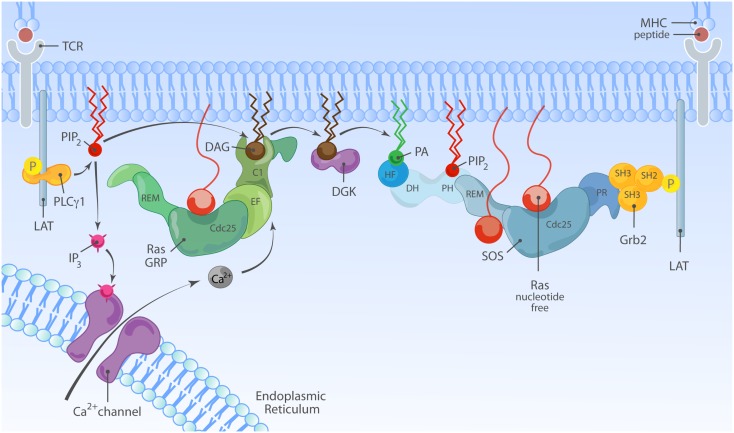
**Model of synergy between RasGRP and SOS in TCR signaling**. TCR stimulation is connected to activation of RasGRP via tyrosine phosphorylation of the adapter molecule LAT and activation of PLCγ1, that metabolizes PIP_2_ into IP_3_ and DAG to trigger two second messenger pathways; Ca^2+^ and DAG. Activated RasGRP can enhance the full activation of SOS by providing Ras·GTP, allosterically activating SOS. In principle, the TCR-LAT-PLCγ1 pathway can also indirectly facilitate SOS activation via DAG; DGK metabolizes DAG and converts it to PA, which is a possible target for the HF and/or PH domains in SOS.

Because of DAG’s prominent role in RasGRP1 and RasGRP3 activation in T- and B-lymphocytes, generation of DAG by PLCγ enzymes, and turnover by DAG kinases (DGKs) should be considered. In agreement with a PLCγ1-DAG-RasGRP1 signaling axis (Figure 5), conditional PLCγ1 knockout mice and RasGRP1-deficient mice share a similar defect in positive selection of thymocytes and ERK activation (18, 119). On the other side of the cycle, DGK’s convert DAG to PA, which is interesting because this would dampen DAG-RasGRP signals but perhaps promote PA-SOS signals. In agreement with a critical role for DGK in dampening RasGRP activity (as well as the activity of other proteins containing DAG-binding C1 domains), deletion of DGKα and DGKγ results in increased incidence of T cell lymphoma (120). In normal T cells, DGK enzymes play a critical role in controlling the balance between activation and anergy or unresponsiveness (121, 122). For a complete review of DAG metabolism and the role of DGK enzymes we refer to Krishna and Zhong (95) in this Research Topic. The role of DAG in RasGRP1 regulation is obvious but may not be exclusive. Non-antigen receptor triggered pathways that are typically not associated with DAG production have been implicated in RasGRP1 membrane localization. Specifically, RasGRP1 but not RasGRP3 signals downstream of the CXCR4 chemokine receptor in thymocytes (123) and a heterodimer of TCR/CXCR4 has been described to recruit the PLC enzymes essential in this pathway (124). How different receptor systems couple to DAG and RasGRP and may be able to synergistically trigger this pathway is an interesting concept for future research.

Whereas RasGRP1 is expressed in various cell lineages (20, 46, 61, 62), it is most abundant in developing thymocytes, which perhaps offers an explanation for the fairly specific thymocyte developmental defect that is observed in RasGRP*1*-deficient animals (18). Reciprocally, RasGRP3 abundance is high in B lymphocytes and RasGRP*3* deficient mice demonstrate B cell defects (99), although there is a role for RasGRP1 in this lineage as well, at least in early B cell subsets (101, 125). The developmental defects in thymocytes lacking RasGRP1 are a consequence of severely impaired positive selection of these cells and biochemically visible through the impaired activation of the ERK kinases (61). A causative link between the impaired RasGRP1-Ras-ERK signaling and defective positive selection has been very nicely provided through the analyses of *ERK-1* and *ERK-2* doubly deficient mice in which the thymocytes also show a positive selection defect (126). Perhaps surprisingly, other RasGEFs, be it of the RasGRP-, Rasgrf-, or SOS-type, do not effectively compensate for the loss of RasGRP1 in thymocytes. The fact that there is only minimal compensation for loss of RasGRP1 coming from RasGRP3 or RasGRP4 (123, 127) makes one wonder about the underlying mechanism. Is it purely the relative abundance of RasGRP1 that bestows its unique function in thymocytes and would expression of RasGRP3 from the RasGRP*1* promoter be able to compensate for the loss of RasGRP1? Or, are there unique biochemical properties in the RasGRP1 protein that are lacking in other RasGEFs?

## Additional Mechanisms of RasGRP1 Regulation

Only a small portion of protein flanks RasGRP1’s catalytic REM-Cdc25 core on the N-terminal side (Figure 2). There is no predicted protein domain in this N-terminal part, but this stretch is either only 9 or 57 amino acids long, depending on the use of an alternative internal start codon in *RasGRP1* or its most N-terminal ATG codon (128). The C-terminus appears far more interesting. Not only does it contain the DAG-binding C1 domain, there are also a pair of EF hands sandwiched between the Cdc25 and C1 domains and a roughly 200-amino acid long C-terminal tail without clear domains except for a leucine zipper motif (51, 129, 130). Significantly, genetic deletion of this 200-amino acid long C-terminal tail reduces the formation of mature thymocytes in RasGRP*1^d/d^* mice (131), thus there are critical regulatory functions encoded on RasGRP1’s C-terminus that are relevant for thymocyte function.

Not all EF hands bind calcium, but RasGRP1 has been reported to bind calcium *in vitro* (51) and the position of the pair of EF hands between the catalytic core and the membrane-recruitment C1 unit is an interesting one. EF hands usually come in pairs and are structures consisting of two α-helices connected by a loop that contain residues such as aspartic acid, which are critical for binding and positioning of a calcium ion. The calcium-binding event induces protein conformational changes through the alteration of the directional vectors of the α-helices (50). It is very possible that calcium binding alters the structural conformation of RasGRP1 and other RasGRP family members. Deducting from cell biological assays, it appears that calcium orchestrates membrane recruitment of RasGRP together with DAG although this may vary from cell to cell type.

Kay and colleagues reported that in a chicken DT40 B cell line, the first EF hand pair enables the recruitment function of a C-terminal PT domain (PM targeting domain), which contains the leucine zipper motif (132). Mutation of the characteristic triplet of negatively charged aspartic acids in the first EF hand results in impaired enrichment of this RasGRP1-EF1μ molecule to the PM, following either BCR or G-protein coupled receptor stimuli. Whereas both of RasGRP1’s EF hands contain very similar triplets of aspartic acids, mutation of these into serine in the second EF hand does not impact the membrane recruitment of the RasGRP1-EF2μ molecule (132). Intriguingly, the contribution of the PT domain toward membrane recruitment appears to differ from cell to cell type; it is substantial in BCR-stimulated B cell lines, very modest in T cell lines, and negligible in fibroblasts (129). It should also be noted that these studies relied on ectopic expression of RasGRP1 that was N-terminally tagged with GFP and that the T and B cells tested in this manner also express endogenous RasGRP1. We will discuss the relevance of overexpression of molecules in the Ras signaling pathway later. The concern of co-expressing a tagged (and mutated) RasGRP1 together with endogenous RasGRP1 is appropriate in light of the predicted leucine zipper. It is possible that the C-terminal leucine zipper motif functions as a RasGRP1 dimerization interface, which would make analysis of the individual contribution of introduced- versus endogenous-RasGRP1 molecules complex. Regardless, the Kay group studies clearly revealed for the first time that calcium-dependent regulation, while incompletely understood, plays an important role in RasGRP1 signaling (Figure 4C). Consistent with the notion of calcium-dependent RasGRP1 regulation, the calcium chelator BAPTA-AM and a calcium channel blocker prevented the appearance of Ras·GTP at the Golgi of activated T-cells in imaging experiments (133) (see below for spatial considerations of Ras activation). In biochemical studies, removal of all free calcium by chelators had only a modest effect on TCR-driven Ras activation (134) and RasGRP1 can activate Ras in T-cells in the absence of free calcium (19), although it is difficult to asses the efficiency of calcium chelation or to determine how much cellular calcium would be needed to couple to RasGRP1. In addition, there is an enrichment of calcium ions near the negatively charged polar headgroups of phospholipids in the PM (135), the localization to which RasGRP1 is recruited via DAG. Perhaps it is the membrane-localized calcium that is most relevant to enhance RasGRP1 function. With these biochemical and cellular experiments in mind, it is interesting to speculate on how the regulation of various of the Lupus-associated *RasGRP1* mRNA splice variants that lack portions of the EF hands may be altered (104).

Are there additional mechanisms of RasGRP membrane recruitment or retention that may rely on protein-protein interactions or phospholipids other than DAG? RasGRP1 can interact with a kinase dead version of PKCθ in transfected cells (100). Similarly, RasGRP1 appears to make contacts with DGKζ (136). It is not clear at this point if these results reflect the common intersection point of DAG or if these are true (perhaps transient) protein–protein interactions between RasGRP1 and PKCθ or DGKζ and what the biological implications of these may be for lymphocytes. SKAPP-55 is a multi-domain adapter molecule that interacts with RasGRP1 in a resting T cell line and SKAPP-55/RasGRP1 interactions become more abundant upon TCR or integrin stimulation (137). The immunological implication of SKAPP55 function and its interaction with RasGRP1 are unclear, both a positive role (138) and a negative role (137) have been proposed. Besides a C-terminal SH3 domain, SKAP-55 contains an N-terminal PH domain (just like SOS). It is highly speculative but interesting to consider that both SOS and RasGRP1 may be regulated by phospholipids like PIP_2_ and PA interacting with PH domains, but that this occurs in an indirect manner for RasGRP1 through its interaction with SKAP-55. Lastly, Cornell and colleagues demonstrated that RasGRP1’s PT domain harbors a basic/hydrophobic cluster of amino acids that is conserved among species and that a protein-purified PT domain can bind to phosphoinositide-containing vesicles (130). Thus, it appears that there will be multiple mechanisms of RasGRP activation and regulation, some perhaps surprisingly similar as for SOS RasGEFs.

## Biochemical Synergy between SOS1 and RasGRP1

When SOS and RasGRP’s are co-expressed in a T cell, TCR stimulation can take two routes to Ras-ERK activation; one through RasGRP and the other through SOS (Figure 5). However, genetic studies in cell lines and mice indicate that RasGRP plays a more dominant role in antigen receptor-stimulated Ras-ERK activation (18, 61, 66, 67, 92, 139). A recent study also reports that SOS1/2 maybe inhibitory for TCR-induced ERK activation in human peripheral T cells (140), although this finding is inconsistent with several other studies showing a positive contribution of SOS in antigen receptor-stimulated ERK activation, both in lymphocyte cell lines and primary mouse and human lymphocytes (20, 65–67, 92). Reduction of SOS expression leads to moderate but consistent ERK activation impairment in human peripheral T cells, mouse DP thymocytes, and DT40 B cell line (20, 66, 67, 92, 139). Furthermore, the ERK activation defect in SOS1^−^2^−^ DT40 cells is most noticeable at low and physiological levels of antigen receptor stimulation, indicating that ranges of stimuli across multiple time points are required to conclusively analyze ERK activation defects (66, 92, 139).

**Figure 6:**
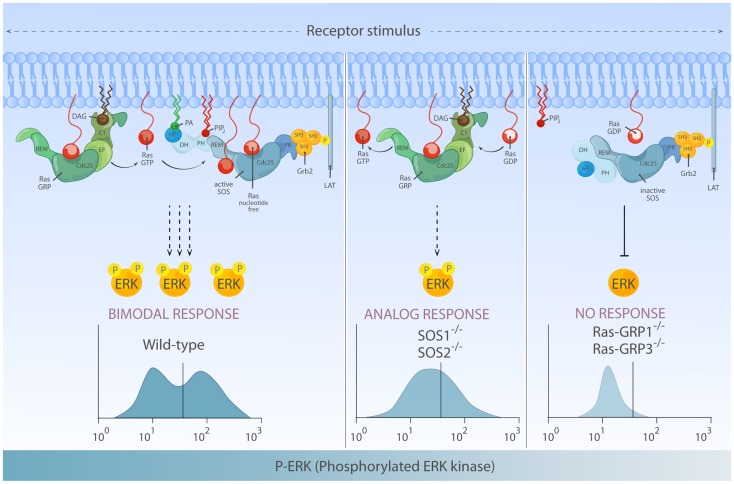
**Differential activation of RasGEF determines the quantity and quality of Ras-ERK output**. Left: full activation of the ERK response requires activation of both RasGRP and SOS and can lead to bimodal (digital) ERK activation patterns. In this mode of signaling, RasGRP activation temporally precedes activation of SOS and provides initial Ras·GTP that primes full activation of SOS. Middle: in the absence of SOS, there is substantial Ras-ERK activation mediated by RasGRP alone, but the ERK activation patterns are analog and therefore differs both quantitatively and qualitatively from ERK signal generated by two RasGEFs in synergy. Right: in lymphocytes, RasGRP plays a dominant role in connecting TCR-Ras-ERK pathway. SOS alone has difficulty to prime its own allosteric activation, which results in a high threshold for Ras-ERK activation.

Interestingly, flow cytometry-based examination of ERK activation for single cells within a population revealed that not only the quantity but also quality of phosphorylated ERK (pERK) output differs depending on RasGEFs connecting stimulated antigen receptor to Ras (66). In the DT40 model B-cell system, the pERK pattern in BCR-stimulated wild-type DT40 cells (co-expressing RasGRP1/3 and SOS1/2) demonstrates a highly thresholded and bimodal/digital pERK pattern. RasGRP1/3 double-deficiency in DT40 cells results in poor pERK response consistent with near abolished ERK activation in RasGRP1-deficient mouse lymphocytes, indicating that RasGRP play a dominant role in ERK regulation (66). In the absence of SOS1/2, RasGRP1/3 can still activate ERK downstream of BCR, albeit at reduced level. More significantly, these flow-based assays show that RasGRP1/3-driven ERK activation gradually increases over time and displays analog/unimodal pERK patterns, but does not yield a bimodal pattern (Figure 6).

Multiple models at different levels of Ras/MAPK signal transduction explain the shaping of digital/bimodal ERK activation, such as Ras nano-clusters (141), dual negative feedback control by SHP-1 (142) or scaffold-mediated signal quality change (143), and subcellular location of cascade activity (144). But, none of pre-existing models explain the change in ERK activation pattern depending on the RasGEFs availability. Based on computer modeling analysis, we hypothesized that the optimal Ras-ERK response observed in wild-type cells co-expressing SOS and RasGRP involved allosteric activation of SOS primed by Ras·GTP produced by RasGRP. To test this hypothesis, we uncoupled the potential positive feedback loop between two RasGEFs by introducing W729E mutation that prevents Ras·GTP binding at the SOS1 allosteric pocket (66, 89, 92). Whereas RasGRP1 is comparably activated (measured by T184 phosphorylation), BCR-induced Ras-ERK response in cells expressing W729E mutant-SOS1 resembles that of SOS1/2-deficient cells [unpublished data (92)]. Which RasGEF generates an initial flux of Ras·GTP priming full activation of SOS? Theoretically, allosteric activating Ras·GTP can come from self (SOS) or from RasGRP. Indeed, HeLa cervix carcinoma cells that do not express RasGRP1 (Roose lab, data not shown) are able to engage the SOS-Ras·GTP-SOS loop in response to EGFR stimulation (91). However, both lymphocyte cell lines and primary lymphocytes lacking RasGRP poorly respond in terms of Ras-ERK activation, indicating that RasGRP plays an essential role in ERK activation by signaling to Ras-ERK but also to Ras-SOS, via an early surge of Ras·GTP allosterically activating SOS (18, 61, 65–67, 92, 139).

## Novel Insights and Puzzles for Thymocyte Selection Signals from Mouse Models Deficient for RasGEFs

In the cellular context where two RasGEFs co-exist, biochemical activation of RasGRP appears temporally ahead of activation of SOS (Roose lab, data not shown). Moreover, whereas SOS requires allosteric activation by Ras·GTP and therefore in a sense relies on RasGRP1 (65), the reverse relationship does not exist: RasGRP1 does not appear to require SOS. These relationships between RasGRP/SOS lead to the hypothesis that the differential fate of thymocytes undergoing selection might be determined by how two RasGEFs are differentially activated upon TCR stimulation (145). This hypothesis was also founded by the observation that positively selected DP thymocytes demonstrate graded (or analog) ERK activation (146, 147). In this model, weak TCR stimulation of positively selected thymocytes sub-optimally phosphorylates LAT, enough to activate PLCγ1-DAG-RasGRP1 pathway and analog ERK signals but without coupling SOS1 membrane recruitment and digital ERK signaling (Figure 6). Genetic support for this model comes from the observation that RasGRP1 is essential for positive selection but is not required for negative selection (61, 67). On the other hand, strong TCR stimulation during negative selection induces extensive LAT phosphorylation, enough to recruit and activate both RasGRP1 and SOS1, and enable allosteric activation of SOS, which generates strong ERK activation characteristic of negatively selected DP thymocytes (66, 147). Whereas this is a plausible model it does not address the question if digital SOS-ERK signals are negative selection cues for thymocytes. In fact, genetic deletion of *ERK-1* and *ERK-2* does not impair negative selection of DP thymocytes at all (148), perhaps arguing that the characteristic ERK activation profiles of thymocytes under negative selection conditions is only a byproduct of a different signal that causes the true negative selection (we will discuss this later). Furthermore, recent studies of a conditional *SOS1* knockout mouse model from Samelson and colleagues provided yet another puzzling insight into the different roles of RasGEFs during T cell development (20, 67), which we will discuss next.

Conditional genetic deletion of *SOS1* in thymocytes, SOS1(T)^−/−^ revealed that SOS1 is dispensable for negative selection, disfavoring the previously mentioned differential-RasGEF-usage model for thymocyte fate decision [reviewed in Ref. (149)]. Instead, SOS1 expression is required for DN stage thymocytes undergoing DN to DP transition. *SOS2* deficiency alone does not significantly affect either positive or negative selection (67). The developmental block in SOS1(T)^−/−^ thymocytes is accounted for by impaired proliferative expansion of DN to DP thymocytes (20). The early developmental defect in SOS1(T)^−/−^ thymocytes can be explained by developmental stage-specific expression profile of different RasGEFs: protein level of SOS1 and RasGRP1 dynamically changes as thymocytes develop. SOS1 protein level is highest in DN thymocytes, while DP thymocytes only express 20% of the SOS1 levels seen in DN thymocytes. RasGRP1 protein level follows the opposite trend: little RasGRP1 is expressed in DN, RasGRP1 is most abundant in DP thymocytes (20, 61). Most puzzling is the finding that combined deletion of RasGRP1/SOS1 impairs negative selection (67). What would be the signaling components regulated by two RasGEFs for negative selection? It is unlikely to be Ras-ERK since negative selection is not affected in H-Ras^S17N^ transgenic nor in *Erk1*^−^*Erk2*^−^ doubly deficient thymocytes, indicating that Ras-ERK activation is dispensable for negative selection (12, 148).

One explanation could be that rather than mediating TCR-sparked responses, both GEFs provide a permissive type of input by sustaining steady-state, basal Ras and Ras-effector levels, as documented in other systems (150, 151). Another explanation may be provided by other functions of RasGEFs in addition to activation of the canonical Ras-ERK pathway. Pharmacological inhibition of the p38 MAPK impairs negative selection in fetal thymic organ culture system (152). Additionally, both Grb2 haploinsufficiency as well as complete Grb2 deletion is concomitant with reduced p38 activation and impaired negative selection (153, 154). We recently uncovered an unanticipated link between SOS and p38 (92). Significantly, SOS1 plays a critical role to connect TCR triggering to p38 activation. By contrast, RasGRP1 plays only a very minor regulatory role in TCR-induced p38 activation in human peripheral T cells and Jurkat cell line and p38 activation is unaffected in thymocytes deficient of RasGRP*1* (92). Surprisingly, SOS1’s role in p38 activation is independent of allosteric activation of SOS or even of any enzymatic activity in SOS1, arguing that this is indeed a non-canonical SOS pathway [discussed in more detail later; (92)].

## Spatial Control of Ras Activation: A Role for Lipid Messengers and GEFs in Compartmentalized Ras Signaling?

Traditionally, Ras activation in leukocytes and other cell types has been intuitively assumed to proceed at the PM based on the notion that Ras activation is bound to happen in close proximity to growth factor or antigen receptor systems that do, in their majority, operate at the cell surface. Early immunocytochemical studies confirmed the predominant presence of Ras at the PM (155–162), lending support to the view that Ras activation proceeds at the PM. However, a diffuse staining of the cytoplasm was apparent in some reports (155, 161, 163), suggesting early on that meaningful amounts of Ras proteins might also be present and signal from internal membranes (endomembranes). The concept that Ras proteins do associate with subcellular membranes was cemented in a series of studies from the 1980s documenting that Ras proteins are subject to a complex series of post-translational modifications that gradually increase their hydrophobicity and effectively govern the association of Ras with cellular membranes [for a review, see (13, 164, 165)]. Recent imaging studies have added a spatial and temporal dimension to this view by showing, firstly, that the stepwise post-translational processing of nascent Ras proteins proceeds at endomembranes *en route* to the PM (163), and second, by disclosing dynamic cycling of the two palmitoylated Ras proteins H-Ras and N-Ras between PM and endomembranes in dependency of their palmitoylation status (166–169). According to this latter “acylation cycle” model, palmitoylation at the Golgi apparatus “traps” H-Ras and N-Ras proteins at endomembranes, tagging them for exocytotic transport and accumulation at the PM. Upon depalmitoylation by the recently characterized acyl protein thioesterase 1 (APT1) (167, 170) and possibly other as yet unidentified depalmitoylating activities, Ras proteins loose their tight and inert binding to the PM, leading to a fast inter-membrane exchange of depalmitoylated Ras and, in consequence, to the tendency to distribute equally to all cellular membrane compartments. One round of the cycle is completed by the renewed palmitoylation of Ras at the Golgi apparatus, a reaction that essentially provides a vectorial component ensuring the predominant localization of Ras at the PM. In contrast to the dynamic palmitoylation-dependent cycling of H-Ras and N-Ras, the non-palmitoylated K-Ras protein is assumed to reside and function largely at the PM, although alternative modes for K-Ras internalization have also been described (171, 172). Knowing this, the intriguing question is whether compartmentalization of Ras activity represents a means of signal diversification in antigen receptor signaling and whether or not second messenger lipids coordinate spatial aspects of Ras activation.

## Imaging Active Ras·GTP in T-Cells

In 2003, Mark Philips and coworkers presented the first of a series of studies that reported for the first time a view of Ras activation in real-time in lymphocytes challenged via the T-cell receptor (133, 173, 174). Ras·GTP visualization was accomplished using a genetically encoded, fluorescent reporter probe composed of EGFP and the Ras-binding domain (RBD) of the Ras-effector c-Raf. EGFP-RBD features several orders of magnitude higher affinity for Ras·GTP versus Ras·GDP causing it to redistribute and illuminate subcellular sites of Ras·GTP accumulation (162, 175, 176). However, levels of endogenous Ras·GTP are too low to be visualized by EGFP-RBD and researchers have been forced to overexpress Ras. Use of EGFP-RBD to image activation of overexpressed Ras in Jurkat T-cells challenged by clustering the CD3ε chain of the TCR alone or in combination with co-stimulatory triggers yielded an unexpected picture: a bimodal pattern of Ras activation consisting of K-Ras activation at the PM followed or paralleled by a more sustained accumulation of N-Ras·GTP at the Golgi apparatus (173). Strikingly, N-Ras became GTP-loaded only at the Golgi despite the fact that it was present in large amounts at the PM, where the same TCR stimulation induced robust GTP loading of K-Ras (174). While the precise mechanisms enabling the TCR to discriminate among Ras isoforms and subcellular platforms of activation are not fully clear, a number of factors involved in spatial control of Ras activation have been characterized. Pharmacological experiments and use of genetically engineered Jurkat lines provided evidence that the delayed Golgi activation of N-Ras occurred by means of a PLCγ1/RasGRP1 pathway acting specifically on Golgi-resident N-Ras (133, 173), whereas SOS and RasGRP1 acted in concert to load K-Ras with GTP at the PM. Intriguingly, the segregation of the Ras·GTP reporter probe to PM versus endomembranes depended on a number of stimulation parameters: first, the strength of TCR stimulation, with low-grade stimulation (achieved by applying CD3 and CD28 cross-linking antibodies at a final concentration of 1 μg/ml) causing the accumulation of the Ras·GTP reporter only at the Golgi apparatus, whilst high-grade stimulation (5 μg/ml) lead to the described b dual activation pattern (133, 173). This distinct activation pattern was attributed to the ability of low-grade TCR signals to engage the Golgi-specific PLCγ1/RasGRP1 pathway but not other pathways targeting K-Ras at the PM (173). Arguing against this scenario, other investigators have reported K-Ras activation in response to anti CD3ε Abs administered at concentrations as low as 0.15 μg/ml (19, 177), suggesting that low-grade TCR signals cannot discriminate between PM and endomembrane Ras-pools or between K-Ras and N-Ras isoforms. Interestingly, non-leukocyte cell lines like COS, MDCK, or HeLa, which do not express RasGRP1 (150, 168) (Roose, unpublished) exhibit the same segregation of EGFP-RBD to the PM and Golgi in response to growth factor stimulation (133, 169, 178, 179), evidencing that mechanisms of endomembrane Ras activation other than the RasGRP pathway do exist. Data from Bastiaens lab illustrate that (overexpressed) Ras·GTP generated at the PM of MDCK cells relocates to endomembranes following its depalmitoylation at the cell surface in the context of the acylation cycle (169, 179). This mode of endomembrane Ras activation may well operate also in T lymphocytes, but this would imply that endomembrane Ras activation should be preceded by a first “wave” of N-Ras activation at the PM, which was not reported in those studies (173, 174). In conclusion, the individual contribution of the two known modes of endomembrane Ras activation in TCR signaling in T lymphocytes still needs to be evaluated in detail.

## The Role of Co-Stimulation

Another parameter that can affect the spatial segregation of Ras·GTP is the nature of the co-stimulus provided along with the CD3-cross-linking Ab. For example, CD28 co-stimulation enhances DAG production in T-cells (121, 180) and this in turn is expected to enhance Ras activation via RasGRP1. CD28 co-stimulation is thus intuitively expected to affect the magnitude and possibly also the location of Ras·GTP formation. Somewhat unexpectedly, therefore, this turned out not to be the case, since co-stimulation with soluble CD28 antibodies does not ostensibly affect Ras·GTP levels and/or Ras·GTP localization (174, 177). Perhaps CD3/CD28 co-stimulation experiments need to be re-evaluated using immobilized rather than soluble Abs for receptor crosslinking (181). Co-stimulation via the lymphocyte function-associated antigen-1 (LFA-1), on the other hand, was reported to stimulate activation of Ras at the PM (174). Interestingly, LFA-1 facilitated Ras·GTP formation by stimulating the generation of DAG at the PM via the sequential action of PLD2 and Phosphatidic acid phosphate (PAP), a pathway that had before been linked to DAG/PA metabolism at the Golgi (182). In agreement with an important role of LFA-1 signals for Ras·GTP formation, co-stimulation via LFA-1 reportedly enhanced Ras·GTP accumulation in response to TCR-clustering (174). In opposition to that scenario, others have not observed an effect of LFA-1 on Ras·GTP levels in T-cells (177). Along the same vein, co-stimulating T-cells via SLAM, a measure that also leads to an enhanced production of DAG in T cells (180) did not further stimulate Ras·GTP production, further indicating that an elevation of DAG levels in response to particular TCR/co-receptor stimulations does not always automatically translate in elevated Ras·GTP levels.

## Endogenous Versus Overexpressed Ras and Other Experimental Considerations

The pioneering imaging studies described above have changed the way we think about Ras activation, away from the traditional, rather unilateral view of “static” Ras proteins acting at the PM to the more dynamic picture that has now emerged and has been delineated in the previous sections. It is, however, important to recall that the experimental approaches that have led to this new conception feature a number of caveats and limitations that should be borne in mind. One limitation is that stimulation with cross-linking antibodies toward the CD3ε chain and various co-receptors, as used for reasons of simplicity in most studies, may not reliably reflect the physiological setting of a T-cell challenged by an antigen-loaded APC. Secondly, overexpression of Ras proteins, as applied in most imaging experiments, is an issue worth considering.

Since Ras activation and trafficking are finely regulated processes it is arduous to judge whether or not images obtained from cells overexpressing Ras proteins do always truly reflect the behavior of endogenous Ras. Evidence arguing that this may indeed be an important fact to bear in mind comes from studies reporting on the subcellular localization of endogenous Ras·GTP in live T cells (177, 183). Visualization of endogenous levels of Ras·GTP in T cells was achieved using refined fluorescent biosensors for Ras·GTP that consisted of three concatenated RBD modules, yielding increased avidity toward Ras·GTP (183), and three EGFP proteins, that conferred threefold higher fluorescence intensity to the probes (177). These probes redistributed only to the PM of PMA or TCR-stimulated Jurkat cells and to the immunological synapse of primary T lymphocytes conjugated to APCs (177, 183), but the probes did not illuminate the Golgi or other endomembranes, in contrast to what was observed in T cells overexpressing H-Ras or N-Ras (133, 174). This remarkable variance in experimental outcome can be interpreted in two ways: first, the trivalent EGFP × 3-RBD × 3 reporter probes do illuminate endogenous Ras·GTP formed at the PM but they are not sensitive enough to visualize Ras·GTP at the Golgi. Since the signals obtained for endogenous Ras·GTP at the PM using the EGFP × 3-RBD × 3 biosensors are clear and well visible, this interpretation would imply that Ras·GTP levels at the Golgi are markedly lower than those at the PM. The alternative explanation is that accumulation of N-Ras·GTP at the Golgi results from perturbances in Ras trafficking, processing, or activation processes as a consequence of Ras overexpression. For example, the reported relocation of GAPs to the cell surface at later stages of TCR signaling for the shutdown of PM Ras signaling (133, 184) could cause a drop in GAP activity at endomembranes that could facilitate increased Ras·GTP loading at the Golgi in a background of Ras overexpression. Also, a sheer increase in the flux of N-Ras through the acylation cycle in Ras overexpressing T-cells is expected to lead to the redistribution of more N-Ras·GTP from the PM to endomembranes. In sum, it is currently difficult to judge whether the observed accumulation of overexpressed N-Ras·GTP at the Golgi is a physiological response of T-cells to antigen stimulation or rather reflects an effect that is only seen with anomalously high levels of Ras.

## Compartmentalization of DAG-RasGRP1 Signals

Given that Ras activation downstream of the activated TCR is largely driven by the concerted action of SOS and RasGRP1 GEFs, can knowledge about the segregation of GEFs and the lipid second messengers that regulates GEF action help us understand the spatial control of Ras activation? The subcellular distribution and TCR-dependent, spatially localized formation of DAG, as the most prominent lipid second messenger involved in the regulation of Ras activity, have been investigated in quite some detail. In addition to its presence at the PM, DAG is present in meaningful amounts at various other subcellular sites including the Golgi apparatus and the nuclear membrane (185, 186). It appears that the sources for these distinct pools of DAG are different. For example, DAG at the Golgi arises largely from Sphingosine metabolism and to some extent also from the sequential action of PLD and PAP on phospholipids (182, 187). PM-located and nuclear DAG is mostly replenished by *de novo* synthesis but is also generated to a variable extent by the action of Phospholipases of various kinds on precursor phospholipids (for comprehensive reviews on DAG metabolism see (187, 188) and in this review issue). Although lymphocytes reportedly have a pool of nuclear DAG, too (186), most attention has been devoted to the PM and Golgi-populations of DAG, since these are, arguably, the two major platforms of TCR signaling. While some subcellular sites, prominently the Golgi apparatus, are rich in steady-state levels of DAG (182), it is generally assumed that DAG-dependent signaling downstream of the TCR involves the *de novo* generation and spatially restricted accumulation of DAG in response to antigen stimulation. Since DAG can directly recruit the Ras activator RasGRP1 it appears reasonable to predict, that domains of DAG formation in response to TCR stimulation should coincide with sites of Ras·GTP accumulation.

Where does TCR-sparked DAG production occur and where within the antigen-stimulated T-cell does DAG accumulate? Several laboratories have imaged DAG in live T-cells using fluorescent reporter probes derived from DAG-binding domains including C1 domains from RasGRP1, PKCθ, or PKD (189–192). Interestingly, C1 domains from RasGRP1 or PKCθ illuminated endomembranes in unstimulated T-cells, suggesting that resting levels of DAG in T-cells are primarily found in that compartment. Upon conjugation with APCs, the same reporter probes relocalized to the IS (190, 191), illustrating that DAG accumulates at the IS. The accumulation of active PLCγ1 (assessed by phosphorylation on Y783) to PM and IS in response to TCR cross-linking or conjugation with APCs (193) is also in line with this view. Consistent with the notion that TCR-activation induces DAG formation/accumulation at the PM, the full-length versions of the DAG-effector proteins PKD and chimaerins accumulate at the PM or IS of TCR-challenged T-cells (194). DGKα and DGKζ, two enzymes that metabolize DAG by converting it to PA, also accumulate at the PM of T-lymphocytes conjugated to antigen-loaded APCs (192, 195), a step proposed to be critical for the spatial confinement of DAG to the IS (196). In the case of RasGRP1, some studies reported exclusive redistribution of RasGRP1 to the PM or IS of T-cells challenged via the TCR (122, 177, 194, 197–200) while others documented TCR-activation dependent accumulation of RasGRP1 at PM and Golgi (133, 147, 174). Importantly, while these considerations may cause the impression that DAG alone determines the subcellular distribution of many of its effector proteins, DAG is likely to be only one of various factors that coincidentally determine the spatial distribution of RasGRP1 and other DAG-target proteins. For example, the DAG-effector PKD features a transient and short-lived recruitment to the IS despite the much more prolonged presence of DAG at the IS (191).

## Compartmentalization of Lipid-SOS Signals?

Recently, the lipid product of PLD, PA, has been reported to recruit SOS via its PH domain, thus providing yet a new link for a lipid messenger and Ras activation. Since PA is found both at the PM and endomembranes (174, 182), mechanisms for the oriented and regulated recruitment of SOS to subcellular membranes must exist. This involves probably the concerted action of PA with other upstream inputs such as PIP_2_, Ras-binding, and Grb2 binding, as described extensively above (see sections on SOS regulation).

Another important second messenger lipid with relevance to SOS-driven Ras activation is the PI3K reaction product phosphatidylinositol-3,4,5 trisphosphate (PIP_3_) (201, 202). The subcellular distribution of PIP_3_ in lymphocytes has been visualized using fluorescent reporter proteins based on the PH-domain of Akt (203–205). These studies reported that PIP_3_ was produced and accumulated at the PM, but in contrast to DAG, PIP_3_ was not restricted to the IS but expanded also to regions outside the IS (203). Indeed, a sustained accumulation of PIP_3_ was even observed at the antisynapse or uropod of the T-cell (204). Remarkably, other upstream modulators or known activators of Ras like ZAP70 and ezrin, respectively, also accumulate at the antipodal pole of conjugated T-cells (206–208). Intriguingly, ezrin is an important co-factor in Sos activation in some systems (207), which raises the intriguing possibility that concerted Sos-dependent Ras activation by means of ezrin and PIP_3_ and subsequent Ras-signaling (to PI3K?) may proceed at the T-cell uropod at later stages of T-cell-APC conjugation.

The subcellular distribution of PIP_3_ in the course of T-cell stimulation is consistent and certainly suggestive of a role of PI3K in the control of Ras activation and/or signal propagation. However, the precise role played by PI3K and its lipid products in Ras activation is an intensively debated, and as yet not settled issue. PI3Ks [refers collectivelly to the four members of the class I family of PI3Ks (209)] were originally described and characterized as effector proteins of Ras, and a large body of experimental evidence [including the recent analysis of transgenic animals expressing PI3K variants that cannot be activated by Ras·GTP (210, 211)] has firmly established the notion that PI3Ks do function downstream of Ras [reviewed in Ref. (212)]. On the other hand, a number of studies has also documented a role for PI3K upstream of Ras (201, 202, 213), indicating that PI3K lipid products could fulfill dual roles as second messengers in the propagation of Ras-sparked signals and as modulators in the (feedback?) control of Ras activation.

How could PI3K lipid products regulate Ras activation in lymphocytes? PIP_3_ interacts physically with the Ras-GAP species GAP1(m) (214) and biochemical evidence for a regulation of Ras-GAP activities by PIP_3_ in leukocytes does exist (202). Beyond this largely unexplored connection with GAP proteins, PIP_3_ interacts with and recruits members of the Tec family of protein kinases, prominently Bruton’s tyrosine kinase, Btk, in B cells and Itk in T-cells (215), via an amino-terminal PH domain (216–218). Tec kinases, in turn, can affect Ras activation in two ways: first, Tec kinases are critically involved in antigen receptor-induced PLCγ activation (219, 220), and defective Tec activation in response to antigen receptor stimulation leads to a number of defects in pathways dependent on DAG/IP3, including PKC and ERK activation (221, 222). The latter finding suggests that Ras activation should also be affected, although this has, to our knowledge, not been directly assessed. Secondly, defects in Tec kinase function cause a decrease in PA levels (223), which could in turn result in diminished Ras·GTP loading via SOS (29). In this regard, it is probably important to consider PIP_3_ in a broader context in conjunction with the fate of its precursor lipid PIP_2_. Beyond serving as a substrate for PI3Ks, PIP_2_ plays a critical function as the substrate of PLCγ enzymes and it is well established that the agonist-evoked activation of PI3K and PLCγ signaling can lead to a marked, acute and probably spatially restricted drop of PIP_2_ levels in leukocytes (224, 225). Since PIP_2_ can modulate Ras activation via the direct, PH-domain dependent interaction with SOS, the concerted and locally confined regulation of the PIP_2_/PIP_3_ ratio is predicted to have a large impact on the activation status of Ras. From a technical point of view, one important challenge for the years to come will be to address this aspect of Ras activation by visualizing PIP_2_ and PIP_3_ simultaneously with Ras·GTP in life cells, an approach that should ideally be expanded to other second messengers involved in the control of RasGEFs.

## A Physiological Role for Compartmentalized Ras Signaling?

Is the segregation of Ras signaling to endomembranes and possibly other subcellular sites an inherent and fundamental component of TCR signaling that provides an additional level of signal diversification? Evidence for a possible physiological role of compartmentalized Ras signaling in T-cell biology comes from provocative data reported by Ed Palmer’s lab arguing that Ras localization and signaling from PM versus endomembranes could be a major fate determinant during thymic T-cell selection (147). Using a collection of agonist ovalbumin (OVA) peptide variants with graded affinities toward the TCR on transgenic OT-I T lymphocytes these investigators observed a distinct compartmentalization of Ras and its downstream effector protein c-Raf (also known as Raf-1) in dependency of agonist strength: in T-cells driven into negative selection by high-affinity antigen peptides Ras and c-Raf distributed largely to the PM whilst positive selecting, low affinity ligands induced a relocation of Ras and Raf to endomembranes. Intriguingly, localization of RasGRP1 followed a similar pattern. At first sight the relocation of Ras signaling to endomembranes by high-affinity ligands in the thymocyte selection model and by low-grade TCR stimulation of Jurkat cells in the study by Perez de Castro et al. (173) may appear hard to reconcile, although it is probably tedious to compare peptide/APC-stimulation of immature double-positive thymocytes with Jurkat cells or primary mature T cells challenged by means of cross-linking Abs. It is also important to note that Ras accumulation at endomembranes, as observed in positively selected thymocytes, must not necessarily reflect high Ras·GTP loading and Ras signaling at that organelle. In this regard, the coincident accumulation of Raf in the Golgi apparatus of positively selected thymocytes may not be a reliable marker for the presence of Ras·GTP as suggested (147). Since only about 3% of c-Raf interacts with Ras·GTP in antigen challenged T-cells at the peak of Ras·GTP formation (226), the observed quantitative relocation of c-Raf to endomembranes is unlikely to result from recruitment by Ras·GTP but could rather argue for the action of a small second messenger molecule in recruiting c-Raf. For example, c-Raf is recruited and activated by PA (227, 228), and thus PA generated by DGK-catalyzed phosphorylation of DAG or by PLD activation downstream of PKC (229, 230) is an attractive candidate in this respect. In sum, the documentation of spatial Ras segregation in the context of thymic selection provides important evidence for a role of compartmentalized Ras signaling in T-cell biology, but we need to understand more about the underlying mechanisms governing spatial control of Ras activity. Moreover, the fact that mice devoid of both palmitoylated Ras variants, H-Ras and N-Ras, live a mostly healthy life (231), have normal T-cell differentiation and feature only relatively minor defects in mature lymphocyte biology (232) evidences that the compartmentalization of Ras signaling to endomembranes is not essential or critically important for TCR-dependent signaling, at least in rodents. Perhaps the ability to compartmentalize Ras signals to endomembranes is part of a signaling repertoire for fine-tuning of TCR responses, the physiological relevance of which has so far escaped our attention.

## SOS1 as a Lipid Regulated Adapter Molecule

Overshadowed by its primary role as a RasGEF in the canonical SOS-Ras pathways, it is relatively underappreciated that SOS1 may function as a scaffold molecule that can potentially sense membrane lipid- and protein-originated signals. Particularly interesting is SOS’s PR C-terminal segment with multiple potential SH3 binding sites (PxxP motifs) and at least four sites that bind to Grb2’s SH3 domain *in vitro* (32, 233). The multiplicity of the SH3 ligand sites in the C-terminus bestows the capacity to interact with more than one interacting partner at any one time. The availability for multiple PxxP motifs opens the possibility for interacting with more than one molecule of Grb2 or other related SOS-interacting SH3-SH2-SH3 adapters such as Grap or Gads. Thus, SOS may function as a scaffold to integrate upstream membrane signals and coordinate activation of multiple downstream pathways.

**Figure 7:**
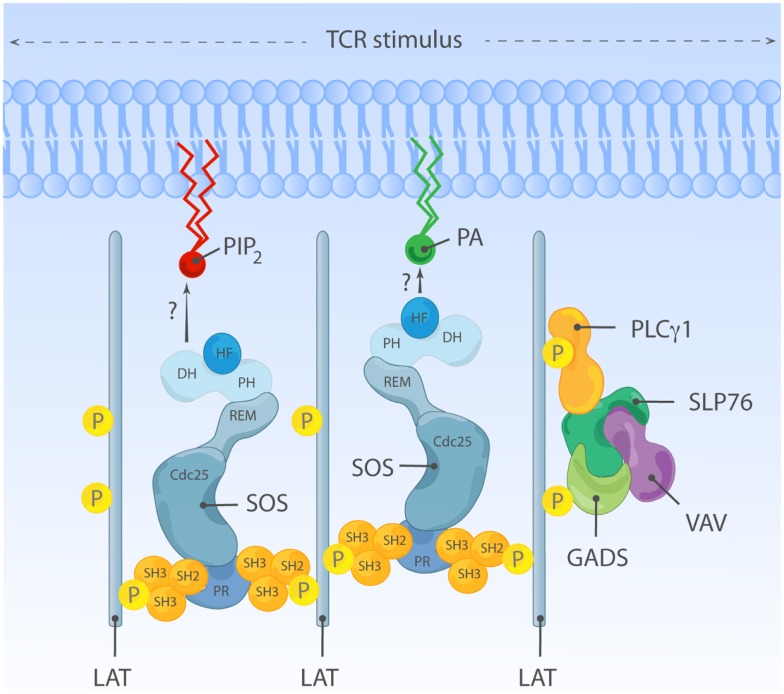
**An adapter function for SOS in oligomeric LAT clusters?** Grb2-SOS complexes can serve as a cytosolic linkers and aggregate multiple LAT molecules and LAT signalosome-constituent proteins together. This SOS-containing complex may facilitate activation of other, non-canonical Ras-ERK signal transduction pathways such as activation of the MAPK p38, perhaps through a Vav-Rac·GTP connection. We found that regulation of p38 is independent of any enzymatic function of SOS, further strengthening the notion that SOS can signal as an adapter to non-canonical pathways in lymphocytes.

Houtman and colleagues actually observed complexes of SOS1 and Grb2 in a 1:2 stoichiometry, particularly when molar concentration of Grb2 is in excess (234). The multivalent interaction between Grb2 and SOS can lead to formation of oligomeric LAT clusters, in this case, SOS-Grb2 complex functions as a cytosolic adapter cross-linking multiple LAT molecules together (234–236) (Figure 7). Expression of PR C-terminal SOS1 fragment in Jurkat cells decreases the size of aggregated LAT clusters and also attenuates weak TCR stimuli-induced calcium flux (234). These observations support the functional existence of SOS-Grb2-LAT clusters, which can facilitate amplification of weak TCR stimulation. SOS can also synergize with LAT clusters by stabilizing LAT signalosome components such as PLCγ1. Upon TCR stimulation, PLCγ1-SH2 is recruited and bound to tyrosine-phosphorylated (Y132; human or Y136; mouse) LAT (237, 238). In addition, the SH3 domain of PLCγ1 directly interacts with PR segments of SOS both *in vitro* and *in vivo*, including in T lymphocytes (35–38). Direct SOS-PLCγ1 binding can promote stable association of PLCγ1 within LAT signalosome by collaborating with SH2-PLCγ1 binding with phospho-LAT. Additionally, direct SOS-PLCγ1 interaction can recruit PLCγ1 to the proximity of its substrate, PIP_2_, which is also a ligand for the HF and PH domains of SOS as described earlier. Thus, it is plausible that LAT and SOS together nucleate a signaling hub in lymphocytes in which many molecules and therefore pathways come together.

Our recent study indicates that SOS1 plays an important adapter function regulating p38 pathway activation independently of SOS1’ catalytic activity (92). In principle, SOS1’s DH domain could act as nucleotide exchange domain in a SOS-Rac-P38 pathway since DH domains are commonly shared structural modules of GEFs regulating Rho family GTPases such as Rac (23, 239). Indeed, SOS has been suggested to operate as a GEF with dual specificity: REM-Cdc25 domains targeting for Ras and DH and PH domain for Rac (240). The latter activity occurs in epithelial cells when SOS1 is coupled to EPS8 and E3b1 co-factors (22, 23, 39). Rac·GTP accumulation is thought to be upstream of classical p38 activation pathway (241, 242). Interestingly, the absence of SOS-1 and -2 profoundly impairs BCR-stimulated Rac·GTP accumulation and p38 activation (92). Combined deficiency of RasGRP-1 and -3 abolishes BCR-induced ERK activation, while its impact on p38 phosphorylation (pT180pY182) is only minimal (92). Unexpectedly, SOS1 versions with either a point mutation (F929A) within Cdc25 that cripples SOS1’s RasGEF function, an allosteric pocket mutation W729E, or a mutation of seven amino acids in the DH domain (LHYFELL → IIIRDII) that would disrupt SOS1’s putative RacGEF activity, all rescue BCR-induced p38 phosphorylation in SOS-deficient DT40 B cells, indicating that enzymatic activity of SOS1 is not required for p38 regulation and SOS1 is functioning as an adapter for p38 activation pathway (92). Thus, whereas the exact nature of SOS1’s adapter function and the potential role of phospholipids binding to SOS1 as an adapter (Figure 7) remain to be further studied, p38 appears to connect to a non-canonical SOS pathway in lymphocytes.

## Concluding Remarks

The need for controlled Ras activation in not only lymphocytes but also in all other cell types is clearly provided by the devastating consequences of aberrant, oncogenic Ras signals in cancer. Not all cell types express both the SOS and RasGRP types of RasGEFs and lymphocytes are perhaps somewhat unique in that these cells have developed an intricate mechanism for sensitive and robust Ras signals via both types of RasGEFs that is still under tight control. We have discussed how membrane recruitment and biochemical activation of the RasGRP and SOS RasGEF is fine-tuned through the concerted input of various mechanisms that include lipid messengers. Future research will undoubtedly further refine the model of Ras activation we sketched here and may reveal how lipid messengers could integrate signals to RasGRP and SOS as adapters in non-canonical pathways that are distinct from Ras.

## Conflict of Interest Statement

The authors declare that the research was conducted in the absence of any commercial or financial relationships that could be construed as a potential conflict of interest.
